# Secretome of Adipose Tissue-Derived Stem Cells (ASCs) as a Novel Trend in Chronic Non-Healing Wounds: An Overview of Experimental In Vitro and In Vivo Studies and Methodological Variables

**DOI:** 10.3390/ijms20153721

**Published:** 2019-07-30

**Authors:** Francesca Lombardi, Paola Palumbo, Francesca Rosaria Augello, Maria Grazia Cifone, Benedetta Cinque, Maurizio Giuliani

**Affiliations:** 1 Department of Life, Health & Environmental Sciences, University of L’Aquila, Building Delta 6, Coppito, 67100 L’Aquila, Italy; 2 Unit of Plastic and reconstructive surgery, Casa di Cura “Di Lorenzo” SrL, Via Vittorio Veneto 37, Avezzano, 67051 L’Aquila, Italy

**Keywords:** chronic non-healing wounds, adipose tissue-derived stem cells, secretome, experimental models

## Abstract

Wound healing is a complex process with a linear development that involves many actors in a multistep timeline commonly divided into four stages: Hemostasis, inflammation, proliferation, and remodeling. Chronic non-healing wounds fail to progress beyond the inflammatory phase, thus precluding the next steps and, ultimately, wound repair. Many intrinsic or extrinsic factors may contribute to such an occurrence, including patient health conditions, age-related diseases, metabolic deficiencies, advanced age, mechanical pressure, and infections. Great interest is being focused on the adipose tissue-derived stem cell’s (ASC) paracrine activity for its potential therapeutic impact on chronic non-healing wounds. In this review, we summarize the results of in vitro and in vivo experimental studies on the pro-wound healing effects of ASC-secretome and/or extracellular vesicles (EVs). To define an overall picture of the available literature data, experimental conditions and applied methodologies are described as well as the in vitro and in vivo models chosen in the reported studies. Even if a comparative analysis of the results obtained by the different groups is challenging due to the large variability of experimental conditions, the available findings are undoubtedly encouraging and fully support the use of cell-free therapies for the treatment of chronic non-healing wounds.

## 1. Introduction

**Figure 1:**
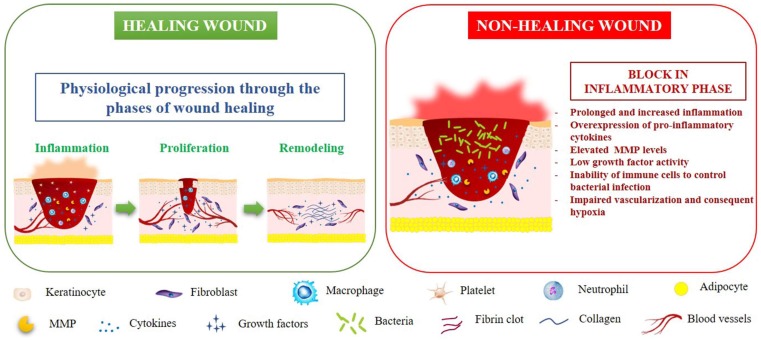
The pathophysiology of healing and non-healing wounds. MMP: matrix metalloproteinase.

Chronic non-healing wounds represent a massive healthcare system problem due to the high incidence and prevalence rate as well as psycho-socio-economic costs [1,2,3]. The surprising increase in the aging population and age-related diseases is one of the principal causes of the high incidence of chronic wounds [4,5,6]. Healthy skin wound healing is a complex and highly regulated process consisting of inflammation, proliferation, matrix formation, and remodeling [7]. When the process is disrupted, chronic non-healing wounds will develop, resulting in an open laceration of varying degrees of severity. In a recent review, Larouche et al. [8] extensively discussed the pathophysiology of both acute and chronic wounds, focusing the attention on the central role played by the immune system in orchestrating every step of the skin healing flux. A molecular pattern with high levels of protease and inflammatory markers, as well as reduced cell proliferation and low growth factor activity is typically associated with chronic wounds. Although the pathophysiology of chronic wounds is still not entirely understood, impaired vascularization and consequent hypoxia, persistent and increasing inflammation, and the ineffectiveness of the immune system in controlling bacterial infections are all crucial factors that negatively affect the physiologic wound closure [9]. Due to the imbalance between pro- and anti-inflammatory signals able to alter the microenvironment, chronic wounds fail to progress beyond the inflammatory phase, which precludes proliferation, matrix deposition, and, ultimately, wound resolution [8]. Hyper-inflammation also leads to an over-expression of metalloproteinases (MMPs), with consequent degradation of growth factors, their receptors, and the provisional extracellular matrix (ECM) essential for cell migration [6]. Also, the lack of growth factors and the accumulation of senescent cells in the injured area slow or block the wound repair process [9]. Figure 1 outlines the pathophysiology of healing and non-healing wounds.

Several factors may affect wound healing and contribute to the pathogenesis of chronic wounds, including infection, ischemia, metabolic conditions, immunosuppression, aging, and radiation [10]. Moreover, genetic differences in immune molecules, including cytokines, receptors, and signal transducers, may influence the inflammation or granulation phases of wound healing [11]. Chronic non-healing wounds may be associated to several different pathological conditions, such as diabetes, venous stasis, and chronic autoimmune diseases, including systemic lupus erythematosus, rheumatoid arthritis, Crohn’s disease, and systemic sclerosis [12,13]. All these conditions are associated to a hyper-inflammatory environment that further impairs physiological healing processes. Ineffectively treated non-healing wounds are often associated with severe complications, including infection, sepsis, and amputation, and can lead to chronic pain, loss of function and mobility, increased morbidity, and mortality. Also, patients with chronic wounds experience social distress, isolation, depression, and anxiety [8]. Current therapeutic interventions, including growth factors, ECM, engineered skin, and negative pressure wound therapy, are often not entirely effective [14,15]. So, due to the significant impact of chronic wounds on the health and quality of life of patients and their families, new and more effective therapies are mandatory.

In this context, increasing attention is focusing on the use of mesenchymal stem cells (MSCs), particularly adipose tissue-derived stem cells (ASCs), to treat chronic non-healing wounds through their trophic, paracrine, and immunomodulatory properties [16,17,18,19,20]. Multipotent adult ASCs could be easily harvested from subcutaneous adipose tissue through minimally invasive surgical procedures, such as liposuction. Of note, the adipose tissue contains higher densities of adult MSCs compared to bone marrow [21,22]. The abundance and accessibility of ASCs together with their multilineage differentiation potential as well as their immunomodulatory properties [23,24] also make them promising candidates for non-healing wound treatment [25,26,27,28,29,30]. However, there are still unresolved critical issues for the definition of effective ASC therapy, which include, among others, the need to standardize methods of isolation, culture, expansion, and characterization of ASCs as well as appropriate quality and safety control for cell-based therapies [31,32,33,34]. Each step of preparation, purification, and expansion of ASCs can, in fact, compromise the quality of the ASCs, leading to progressive cellular senescence, genomic instability, reduction of the level of proliferation, and gradual loss of differentiative potential [35,36]. Other factors that can strongly affect the safety, quality, and properties of ASCs include either the anatomical site of adipose tissue removal/lipoaspirate or the general conditions of the donor (age, body mass index, age-related chronic diseases) [22]. Moreover, several studies have revealed that the implantation time of ASCs is usually too short to have an effective impact [37]. In this context, ASC paracrine activity, through the secretion of a plethora of bioactive factors, was suggested to have the most prominent therapeutic impact in several conditions, including wound healing [38,39,40,41,42]. In this scenario, the study of ASC-conditioned medium (ASC-CM) (also known as ASC-secretome) has opened the way for the development of new cell-free therapies for tissue repair and regeneration [43,44,45,46,47,48]. Like total secretome, its vesicular fraction (the so-called extracellular vesicles, EVs), able to transfer active cargoes between the cells, is thought to exert biological activity similar to ASCs, thus representing another promising novel therapy against non-healing wounds [38,49,50,51].

According to our knowledge, there are no published studies aimed at assessing the effect of the soluble fraction of EV-depleted ASC-secretome on in vitro or in vivo wound healing models. Most in vitro or in vivo studies analyzed the pro-wound healing actions of whole ASC-secretome and its EV fraction. However, it might be of interest to study the specific effect of the different fractions of the secretome, especially in light of the findings of Mitchell et al. [52], which suggested that the EV fraction and soluble molecules within ASC-secretome act in a synergic manner to promote muscle regeneration. Of note, the authors reported that the isolated EV fraction and the EV-depleted soluble fraction showed a distinct spectrum of proteins, as evidenced by mass spectrometry and bioinformatic analysis. In this review, we collected the results of experimental studies aimed at assessing both the beneficial effects of ASC-secretome and EVs on the cutaneous wound healing process and the underlying biomolecular mechanisms. In particular, we focused our attention on the methodologies applied as well as on the in vitro and in vivo models chosen in the described studies to try to define an overall picture of the available literature data.

## 2. Effects of ASC-Conditioned Medium/Secretome on Wound Healing

The term secretome refers to a complex set of multiple soluble factors, such as cytokines, chemokines, and growth factors as well as vesicles, secreted virtually by all types of living cells, including MSCs, in the extracellular space [38,53,54,55]. Several studies have focused on the potential effects of the paracrine activity of ASCs, with the purpose of defining new cell-free therapies for the treatment of chronic wounds.

### 2.1. In Vitro Studies

In an attempt to explore the contribution of ASC-CM on the wound healing process, in 2007, Kim et al. [56] reported that ASC-CM enhanced in a dose-dependent manner the proliferation of primary human dermal fibroblasts (HDFs) as well as their migration on scratched monolayers and type I collagen secretion by regulating the mRNA levels of ECM proteins. In this work, ASCs, after isolation from human subcutaneous adipose tissue samples, were cultured and expanded in control medium of Dulbecco’s Modified Eagle Medium (DMEM) with 10% fetal bovine serum (FBS) and used at passage 1–5. After culturing ASCs for 72 h in serum-free DMEM/F12, CM was collected, centrifuged at 300× *g* for 5 min, and filtered through a 0.22 µm syringe filter. CM was then applied to HDFs at varying dilution folds (0%, 10%, 50% and 100%) in DMEM/F12 with 2% FBS. Using the same cellular model, Zhao et al. [57] showed that CM collected from ASCs (at passage 3–7) cultured for an additional 48 h in serum-free DMEM/F12, at 50% dilution, promoted the proliferation and migration of HDFs. To analyze the effects of ASC-CM on ASCs, HDFs, normal primary human umbilical vein endothelial cells (HUVECs), and human keratinocytes, Kober et al. [58] used CM collected from stem cell-enriched stromal vascular fraction (SVF) cultured in either FBS-free or FBS-supplemented DMEM for 20 h. ASC-CM was used at a 1:1 dilution on cell cultures. In these conditions, the authors obtained a stimulatory effect just on the proliferation of ASCs at 48 and 72 h. No effect was observed on HDF and HUVEC growth, while the proliferation of keratinocytes was significantly reduced at 72 h. Moreover, cell migration tested with in vitro scratch assays at 24 h was not affected by the addition of ASC-CM. Collawn et al. [59] showed that addition of ASC-CM in a 3-D skin raft model produced an acceleration of wound repair similar to that observed with ASCs. ASC-CM was collected from ASC of passage 0 or 1, after 3 days in DMEM without FBS. CM was centrifuged at 1000 rpm for 5 min. The supernatant was recovered without filtration and stored at −20 °C. CM was added, at a 1:1 dilution, to the raft cultures immediately after a laser-induced injury. Seo et al. [60] reported that ASC-CM, similarly to ASCs, was able to stimulate the proliferation, migration, and invasion abilities of HUVECs. HaCaT cell proliferation and migration and vascular endothelial growth factor (VEGF) secretion were also enhanced after incubation with ASC-CM. In this study, ASC-CM was prepared by incubating ASCs with serum-free DMEM for 24 h. No details on the concentration of the CM used were provided by the authors. In another study [61], ASC-CM, previously collected from ASCs cultured for 48 h in serum-free MSC basal medium, was shown to stimulate HDF migration in vitro by 43% when compared to untreated cells as analyzed through a scratch and electric cell-substrate impedance assays. To investigate the underlying molecular mechanisms driving the therapeutic impact of the ASC-secretome, Park et al. [46] used CM samples collected from cultured ASCs previously isolated from adipose tissues of breast cancer patients. Once 70% confluence was reached in complete serum-containing medium, ASCs were incubated in serum-free DMEM for 48 h. CM was then collected, centrifuged twice at 1500 rpm for 3 min to eliminate cell debris, and filtered through a 0.45 µm syringe filter. CM was then mixed with 3 vol of 100% ethanol and incubated for 1 h at −20 °C, after which the ethanol-containing medium was centrifuged at 2000 rpm for 15 min at 4 °C. The pellet was washed with 70% ethanol and centrifuged at 1500 rpm for 15 min at 4 °C. The secretome containing pellet was resuspended in distilled water and used immediately or stored at −80 °C. The addition of so-prepared secretome at 10 µg/mL on HDFs, normal adult human primary epidermal keratinocytes (HEKa), or HUVECs was able to potentiate cell proliferation, migration, and invasion activities as assayed through conventional in vitro tests. Kim et al. [62] recently reported the effects of ASC-CM (0.3 µg/mL) from 3D- or 2D-cultures on both HDF and HaCaT cell proliferation (12 h) and migration (0, 12, and 24 h) through MTT (3-(4,5-dimethyl-2-thiazyl)-2,5-diphenyl-2*H*-tetrazolium bromide) and scratch assays, respectively. ASCs at passage 3–7 were cultured in DMEM with 10% FBS until the cells reached 80% confluence, after which the culture medium was replaced by serum-free DMEM and the cells were incubated for an additional 48 h in 2D-culture or 3D-polystyrene scaffolds. The CM was then collected, centrifuged at 415 *g* for 5 min, and then filtered through a 0.22 µm syringe filter. The results showed that 3D-ASC-CM exhibited significantly greater effects on the proliferation of HaCaT cells than 2D-ASC-CM. On the other hand, no difference was observed between 2D- and 3D-ASC-CM on the HDF proliferation rate. The scratch assay evidenced that both 2D- and 3D-ASC-CM accelerated scratched monostrate closure, with 3D-ASC-CM being more effective on cell migration at 24 h than 2D-ASC-CM. Of note, proteomic analysis of the ASC-CMs revealed that collagens and actin were highly expressed in the 3D-culture system, while chitinase 3-like 1, a tissue inhibitor of metalloproteinases, and galectin-1, which play important roles in the wound healing process, were specifically expressed only in 3D-ASC-CM. To evaluate the growth factor profile in ASC-CM, CM samples collected by ASC cultures at passage 3 were analyzed through the enzyme linked immunosorbent assay (ELISA) method [63]. The medium was collected and centrifuged at 1600 rpm for 5 min and then filtered by a 0.22 µm filter unit. The results suggested a higher concentration level of fibroblast growth factor (FGF), known to be implicated in wound healing and regeneration. Stojanovic and Najman [64] recently compared the immunomodulatory and wound healing potential of CMs collected from stem cells previously isolated from adipose tissue or lipoma and cultured in standard conditions (DMEM with 10% FBS) for three days before passage 2. The effects of CMs were analyzed on RAW 264.7 and L929 cell lines as in vitro models for macrophages and fibroblasts, respectively. The authors concluded that both CMs led to functional activation of macrophages after 48 h of incubation, with a slightly more pronounced effect of CM derived from lipoma-ASCs, while both CMs induced similar changes in macrophage polarization towards a reparative M2 phenotype. Gene expression analyses showed that tumor necrosis factor (TNF) expression level was decreased while interleukin-10 (IL-10) was increased in macrophages cultured with each CM, compared to standard medium. Both CMs were also able to enhance wound closure and cell migration in vitro comparably. Table 1 summarizes the studies on the effect of ASC-CM/secretome on in vitro wound healing models.

### 2.2. In Vivo Experimental Studies

To explore the paracrine functions of ASCs on the regeneration of wounded skin, Heo et al. [65] studied the effects of TNF-α-activated ASC-CM on tissue regeneration using a rat excisional wound model. To prepare ASC-CM, the authors used the method of Lee et al. [66]. Briefly, isolated ASCs were cultured in the complete medium until sub-confluence was reached and then, after removing the medium, were incubated for an additional 48 h in minimum essential medium (MEM)-α in the absence or presence of carrier-free human recombinant TNF-α (10 ng/mL). CM was collected, centrifuged at 3000 rpm for 10 min to remove cell debris, filtered through a 0.2 µm filter, and desalted using a hydrophilic-lipophilic balance extraction column. Excisional wounds were treated daily up to day 12 with ASC-CM or TNF-α ASC-CM. Wound closure, angiogenesis, proliferation, and macrophage infiltration were all stimulated by TNF-α-activated ASC-CM to a higher extent when compared to control ASC-CM. Moreover, the involvement of IL-6 and IL-8 in the pro-healing effects of TNF-α-activated ASC-CM was also suggested. Using the rat skin excisional wound-healing model, Su et al. [67] showed that fibrous scaffolds could potentiate the pro-healing paracrine functions of ASCs. The authors cultured ASCs with different substrates for 1 to 5 days. To collect CM, ASCs were seeded on 24 wells or 1.9 cm^2^ scaffolds and firstly cultured in complete MEM-α medium for 12 h, then washed and supplemented with FBS-free MEM-α medium. After 24 h, the CM was collected, centrifuged at 5000 rpm to remove cell debris, concentrated approximately 30 times by ultrafiltration with a 3 kDa molecular weight cut-off filter, and used for in vivo experiments. A total of 200 µL CM were administered to animals, of which 150 µL was injected subcutaneously around the lesion and 50 µL was applied to the bed of the wound. Digital images of each wound were taken at day 0, 4, and 7, and the wound area was measured by image software. The results suggested that the secretome of scaffold-cultured ASCs cultured has higher levels of cytokines with anti-inflammatory and pro-angiogenic functions with greater therapeutic effects in the rat skin excisional healing model. In the already mentioned article of Park et al. [46], the authors investigated whether ASC-CM could accelerate cutaneous wound healing also in vivo using nude mice after creating full-thickness excisional skin wounds bilaterally on the dorsal surface. After topical application of 30 µg/mL ASC-CM, obtained, as yet described, from ASCs isolated from adipose tissue of breast cancer patients, the lesions were covered with a transparent dressing. The evaluation of lesions at day 0, 2, 5, and 10 indicated that ASC-secretome treatment was able to stimulate skin thickening, angiogenesis, and recruitment of immune cells, thus accelerating the wound healing process. Using a rat model of an ischemic wound (Fisher 344 rats, full-thickness excisional wounds created with a 6 mm punch biopsy), Cooper et al. [61] reported that ASC-CM collected from ASCs cultured for 48 h in serum-free MSC basal medium and applied at 20 µL to each wound daily significantly accelerated ischemic wound closure compared to unconditioned medium. Irons et al. [68] recently showed that after topical application of CM collected from ASCs isolated from the gluteal region of Yorkshire pigs, the diabetic wound healing in the same animals was significantly enhanced, with concomitant increased angiogenesis and immunomodulation. ASCs were cultured with M-199 medium with 10% FBS and then harvested at 90% confluence, and the cell pellets were resuspended with phosphate buffered solution (PBS). CM preparation details are not specified in the article. Wounds designated for CM therapy received topical application of 2 mL of ASC-CM every 3 days. The progression of wound healing was analyzed at day 10, 15, 20, and 28. The wounds topically treated with ASC-CM displayed a significant increase in the percentage of wound closure compared with control wounds at various time points throughout the study. The results from the histologic, mRNA, and protein analyses suggested increased angiogenesis and a reduced inflammatory profile after topical treatment with ASC-CM. Table 2 summarizes the studies on the effect of ASC-CM/secretome on in vivo wound healing models.

## 3. Effects of ASC-Secreted Extracellular Vesicles on Wound Healing

EVs can be separated into three major classes based on their biogenesis: Exosomes, microvesicles, and apoptotic bodies [49,69]. Exosomes (Exos) have a diameter in the range between 20 and 150 nm, are formed within endosomal compartments, and are secreted in the extracellular medium after the fusion of multivesicular bodies with the plasma membrane. Exos have been reported to possess similar functional properties to MSCs from which they derived and exert a crucial role in cell–cell communication [70,71]. Microvesicles (MVs) are generally larger (100–1000 nm) and are formed by direct budding of the plasma membrane. Apoptotic bodies, with a diameter between 50 nm and 5 µm, are released through membrane blebbing upon programmed cell death. Despite the above classification, it is not easy to define with absolute certainty the type of vesicle, even considering that the size range is partly overlapping. Besides, there is also a lack of specific markers and difficulty in accurately defining the biogenesis pathways of vesicles. Thus, the collective term “extracellular vesicles” is recommended [69,72].

### 3.1. In Vitro Studies

Lopatina et al. [73] reported findings indicating that ASC-EVs may contribute to the ASC-induced angiogenesis on human microvascular endothelial cells (HMEC) and suggested that platelet- derived factor growth (PDFG) may trigger the release of EVs with enhanced angiogenic potential. The effects of ASC-EVs were studied in vitro on HMEC proliferation, invasion, and vessel formation using standard methods. ASC-EVs were collected from ASC-CM in basal conditions and after stem cell treatment with growth factors. Human ASCs were acquired and cultured in complete MSC growth medium. For the collection of EVs from the CM, ASCs at passage 4–6 were cultured for 24 h without FBS. After centrifugation at 3000 g for 30 min to remove cell debris, cell-free supernatants were submitted to differential ultracentrifugation at 10,000 *g* or 100,000 *g* for 3 h at 4 °C and EVs were analyzed by NanoSight, showing a mean size of 230 to 250 nm, regardless of the speed of ultracentrifugation. After ASC treatment with PDGF, the secretion of smaller EVs was observed. Also, in basal conditions, ASCs were reported to release 1 × 10^5^ ± 1 × 10^3^ EVs/cell while, when stimulated with PDGF, the amount of EVs released by ASCs significantly increased. Of note, the authors reported no difference in biological activity between fresh or −80 °C stored EVs. Internalization of ASC-Exos by primary HDF was investigated by Hu et al. [74] through in vitro tracking experiments in which labeled ASC-Exos were incubated with HDFs for 24 h, and their cellular uptake was evaluated by fluorescence microscopy. Exos were isolated from the CM of ASCs cultured in serum-free medium for 24 h. After cell debris removal by centrifugation at 3000× *g* for 15 min, the supernatant was filtered with a 0.22 µm filter, concentrated using a 100 kDa molecular weight filter device, and incubated with a commercial Exo precipitation solution for 12 h. Exo pellets were then resuspended in PBS, filtered again with a 0.22 µm filter, and observed for morphology with TEM. The size, concentration, and particle size distribution were characterized by a NanoSight system and Nanoparticle Tracking Analysis (NTA) software while the protein markers cluster of differentiation CD9 and CD63 were analyzed by western blotting. The authors showed evidence that ASC-Exos entered into the cytoplasm of fibroblasts, mainly localizing to the perinuclear region. Moreover, in the same article, the stimulating effects of ASC-Exos at 0, 25, 50, and 100 µg/mL on HDF migration through scratch closure test and transwell assay as well as cell proliferation and collagen synthesis in vitro were also reported. Cooper et al. [61], in addition to investigating the effects of ASC-CM described in Section 2.1, using the in vitro HDF model also evaluated the effectiveness of ASC-Exos in accelerating the wound healing process. Exos, quantitated using a NanoSight system, showed an average size of 90 to 110 nm at a concentration of 1.1 × 10^8^/mL of CM derived from 10^6^ ASCs. In particular, the authors reported that a purified population of ASC-Exos (10 or 20 µg/mL) was able to enhance the cell migration rate after wounding in cultured HDFs. To analyze the mechanisms behind the effects of ASC-Exos in the promotion of angiogenesis on HUVECs in normoxic and hypoxic conditions, Xue et al. [75] used Exos isolated with a complex and articulated method. After the expansion step, ASCs were cultured with DMEM/F12 medium without FBS for 36 to 48 h before extraction of Exos. After centrifugation at 1500× *g* for 10 min to remove cell debris, the culture medium was filtered with a 0.22 µm filter to remove residual cell debris and large vesicles. The filtrate was then ultrafiltrated at a 100,000 molecular weight under nitrogen pressurization. Exos remaining on the ultrafiltration membrane were thus washed, resubmitted to two steps of nitrogen pressurization, and ultracentrifuged at 700,000× *g* at 4 °C for 40 min. Pelleted Exos were resuspended in DMEM and filtered again with a 0.2 µm filter. TEM observation showed that a large number of Exos of 30 to 100 nm could be extracted as described above. EV-protein markers, such as heat shock proteins HSP70, HSP90, and CD63, were analyzed by western blotting. Exo uptake by HUVECs was verified by labeling Exos with Dil (1′-Dioctadecyl-3,3,3′,3-tetramethylindocarbocyanine perchlorate) and by observation of the cells under a fluorescence microscope after 24 h of incubation with labeled Exos. Of note, the rate of ASC-Exo uptake of the hypoxic Exos was higher when compared to the normoxic group. In addition, the angiogenic activity of HUVECs was significantly enhanced with Exos from the hypoxic group compared with those from the normoxic group. Accordingly, angiogenesis-stimulating genes were also significantly upregulated with Exos from hypoxic conditions while angiogenesis inhibitory genes were downregulated. The group of Ferreira et al. [76] explored the ability of ASC-EVs to accelerate in vitro migration, proliferation, and protein kinase B (Akt) pathway activation in primary human keratinocytes and HDFs. ASCs isolated from human subcutaneous adipose tissue, at passage 4 or 5, were incubated with serum-free DMEM for 48 h at 37 °C, after which CM was submitted to multiple centrifugation steps at 4 °C; that is, 300× *g* for 7 min, 1000× *g* for 15 min, and 100,000× *g* for 70 min. The obtained pellet was reported to be composed of different EV populations as revealed by a NanoSight system using Nanoparticle tracking analysis (NTA) software. The average diameter of EVs prepared as described was 135 nm, and their concentration was 1.89 × 10^8^ particle/mL. The authors reported that HDFs and keratinocytes exposed to ASC-EVs prepared as described, at the concentration of 3.16 × 10^7^/mL for 8 days, showed a higher proliferation and migration rate. Similarly, the migration ability of both cell types was also enhanced by administration of ASC-EVs at the same concentration as analyzed through the scratch wound healing assay at 12 and 24 h. Exposure of both cell types to ASC-EVs was also associated with higher levels of phosphorylated Akt and increased activity of the Akt pathway, one of the major biochemical pathways regulating the migration of epithelial cells [77]. More recently, Zhang et al. [78] also showed evidence of the involvement of phosphoinositide 3-kinase (PI3K)/Akt signaling in the promoting effect of ASC-Exos of wound healing. Exos were isolated from ASC-CM as follows. ASCs, at passage 3–5, were cultured in DMEM/F12 with 10% FBS. When cell confluence reached 80%, fresh medium with Exo-free 10% FBS (after ultracentrifugation at 120,000× *g* for 16 h at 4 °C) was added for an additional 48 h culture. To eliminate cell debris, CM was harvested at 4 °C with three centrifugation steps (300× *g* for 10 min, 2000× *g* for 10 min, 10,000× *g* for 30 min). Exos were collected from the supernatant by centrifugation at 100,000× *g* for 70 min at 4 °C. The pellets were resuspended in PBS, and an additional final ultracentrifugation step was performed at 100,000× *g* for 70 min. Thus, the pellets from 100 mL of CM were finally resuspended in 400 µL of PBS. Exo ultrastructure was verified by transmission electron microscopy (TEM) and the size distribution assessed with the ZetaView system. The Exo protein markers, CD63 and HSP70, were analyzed by western blotting. To explore the effects on cell proliferation and migration in vitro, HDFs were treated with 0, 25, 50, and 100 µg/mL ASC-Exos for different times. Cell growth was analyzed at 6, 12, 24, and 48 h while a cell migration assay was performed up to 24 h. The results indicated that ASC-Exos were internalized by HDFs, which registered significant dose-dependent increases in cell proliferation and migration compared to control cells. The treatment also led to higher levels of type-1 and type-3 collagens by upregulating the PI3K/Akt signaling pathway. A recent article of Ren et al. [79] also reported that microvesicles (MVs) derived from ASCs were able to stimulate HUVECs, HaCaT cells, and primary human foreskin fibroblasts due to the activation of the Akt and extracellular signal-regulated kinase (ERK) signaling pathways. In this work, ASCs, previously isolated from subcutaneous fat specimens, were used at passage 2–7 for the collection of microvesicles. Briefly, ASC culture supernatants were collected every 72 h, starting at passage 2 until passage 7, when the cells reached 80% to 90% confluence. MVs were isolated from CM after centrifugation at 1000× *g* for 10 min and 4000× *g* for 30 min, concentration using a 100 kDa molecular weight Amicon filter device, and additional centrifugation at 13,000× *g* for 1 h. MVs, as verified by TEM and dynamic light scattering, were approximately 3 µg/million cells and showed an average size of 235.5 ± 15.4 (range 90–900 nm). ASC-Exos (70–150 nm) have recently been reported to be enriched by microRNAs with anti-cellular aging and regenerative potential and to play a pro-survival role in HDFs by stimulating the cell cycle at a concentration of 5 or 10 µg/mL [80]. After co-incubation for 24 h with PKH26-labeled ASC-Exos, red fluorescence was localized within the cytosol of HDFs, thus confirming the Exo internalization. Li et al. [81] suggested that the pro-healing action of ASC-Exos could be mediated by overexpression of nuclear factor-E2-related factor 2 (Nrf2), a transcription factor with a protective role against oxidative stress [82]. The authors isolated Exos from the CM of ASCs cultured in EGM-2MV medium deprived of FBS and supplemented with serum replacement solution for an additional 24 h. CM was centrifuged at 300× *g* for 10 min and 2000× *g* for 10 min to remove cell debris. Supernatant mixed with a commercial exosome precipitation solution and incubated at 4 °C for 12 h was then centrifuged at 1500× *g* for 30 min to obtain a pellet containing Exos. A Zetasider Nano analysis system revealed a diameter of approximately 100 nm. The addition of ASC-Exo at 50 µg/mL to cultures of endothelial progenitor cells isolated from the peripheral blood of healthy subjects or patients with diabetes mellitus led to a significant increase in cell viability and tube formation ability and an improvement of senescence marker protein SMP30 and VEGF levels. On the other hand, levels of ROS and inflammatory cytokines were significantly decreased by treatment with ASC-Exos. Using HaCaT cells exposed to hydrogen peroxide (H_2_O_2_) for the establishment of a skin lesion model, Ma et al. [83] explored the effects of Exos derived from human facial adipose tissue on cell proliferation assessed by the cell counting kit (CCK)-8 assay, and the migration rate analyzed with standard scratch wound healing and transwell assays. Exos were extracted from 24 h ASC-CM using a commercial exosome isolation reagent, characterized with TEM for the morphology and size (30–100 nm). Western blotting was used to detect CD63 and CD9 expression levels. Cell proliferation was assessed 1, 2, and 3 days after treatment. After in vitro wounding, images for each scratch were observed by optical microscope and photographs were captured at different time intervals for quantitative analysis. For the transwell assay, the number of migrated cells was counted after 24 h incubation. The concentration of Exo suspension added to cell cultures was not specified in the article. The authors showed evidence that ASC-Exos were able to prompt proliferation and migration of HaCaT exposed to H_2_O_2_ by repressing apoptosis through inhibition of Bcl-2-associated X protein (Bax) increment and B-cell leukemia/lymphoma-2 (Bcl-2) reduction in turn induced by oxidative damage. Of note, the positive role of ASC-Exos appeared to be mediated by wingless/integrated (Wnt)/β-catenin signaling, a pathway implicated in the cutaneous healing process [84]. Table 3 summarizes studies on the effect of ASC-secreted extracellular vesicles on in vitro wound healing models.

### 3.2. In Vivo Studies

The pro-healing effects of ASC-Exos on a mouse skin incision model have been reported [74,78]. Isolated ASCs were cultured in serum-free medium for 24 h to collect CM. After the removal of cell debris by centrifugation at 3000× *g* for 15 min, CM was filtered through a 0.22 µm filter, the supernatant was concentrated with a 100 kDa molecular weight Amicon filter device and then incubated with commercial exosome precipitation solution. After being resuspended, exosomes were further passed through a 0.22 µm filter and diluted with PBS. Purified Exos ranged between 30 and 100 nm in diameter. The Balb/c mice with skin wounds were treated with 200 µg ASC-Exos/200 µL PBS through a subcutaneous or intravenous injection. For in vivo tracking experiments, ASC-Exos were labeled with 1,1′-Dioctadecyl-3,3,3′,3′-Tetramethylindotricarbocyanine Iodide (DIR) and the tail was intravenously injected to monitor Exo migration and distribution by observing animals under a bioluminescence system at day 1, 3, 7, 14, and 21. Of note, bioluminescence imaging showed that Exos accumulated in the wounded area at 7 days and could be detected until day 21 while no fluorescence could be detected in control animals injected with dye alone. Wound closure was accelerated by ASC-Exo treatment when compared to untreated or PBS-treated controls. Of interest, intravenous injection of Exos was significantly more effective in promoting wound repair than local injection. The Wistar rat excisional wound-splinting model was used by the group of Ferreira et al. [76] to evaluate the ability of ASC-EVs to promote skin repair. ASCs isolated from human subcutaneous adipose tissue, after expansion in complete medium, were incubated with serum-free DMEM for an additional 48 h, after which the CM was submitted to successive centrifugation steps, as yet described in Section 3.1. For animal treatments, a hydroxyethyl cellulose aqueous gel used to deliver EV suspension (1.9 × 10^8^ EVs) was aseptically applied on the lesions. Wound area was measured at 7, 14, and 21 days after the excision. The results suggested that topical application of ASC-EVs induced a wound healing acceleration, which was comparable to that reported by other authors with more invasive treatments [85]. In addition to what was already mentioned in Section 3.1 for in vitro studies, Ren et al. [79] also investigated the ability of MVs derived from ASCs to promote skin wound healing in vivo. The BALB/c mice excisional wound model was used in order to evaluate the pro-healing efficacy of the administration of ASC-MVs. The wounds were subcutaneously injected with 50 µg ASC-MVs (prepared as previously described in Section 3.1) at five sites after the lesions were created. Digital images were captured at day 0, 3, 7, 10, and 13 and the wound areas were measured using image software. In vivo tracking experiments, performed to control the distribution of injected MVs at 50 µg/mL, showed that ASC-MVs could exist for 15 days in the damaged skin area. The results indicated that ASC-MVs significantly accelerated wound repair and collagen deposition, thus promoting wound healing. Table 4 summarizes the studies on the effect of ASC-secreted extracellular vesicles on in vivo wound healing models.

## 4. Conclusions

**Figure 2:**
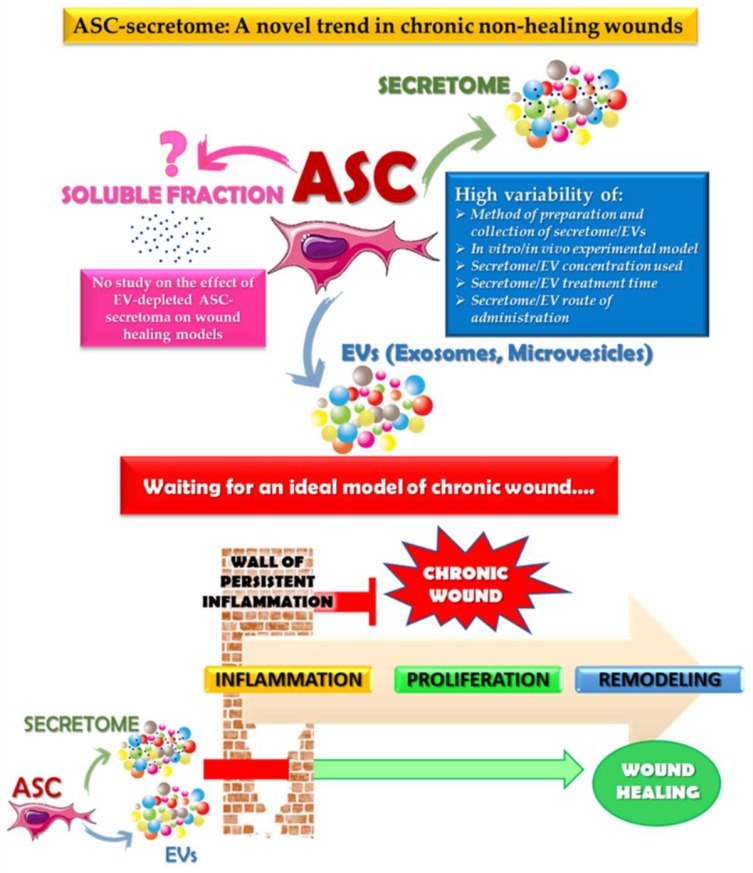
Schematic representation of the issues related to the ASC cell-free treatment approaches used in the wound-healing experimental studies (described in the text). Identifying an ideal chronic wound model would help to assess the benefits of cell-free ASC-CM or ASC-EV therapy. ASC: adipose tissue-derived cells; CM: conditioned medium; EV: extracellular vesicle.

Overall, the in vitro studies examined in the present review, aimed at demonstrating the accelerating effects of ASC-CM or ASC-EVs on the wound healing process, were conducted mainly on cell lines or primary cultures of fibroblasts, keratinocytes, or endothelial cells. Cell proliferation, scratch assay, transwell migration test, and tube formation assay were the most commonly used tests. On the preparation of ASCs, CM, or EVs, a picture of wide variability emerges as well as in the treatment carried out on the different target cell types, both in terms of CM dilution or EV amount and incubation times. Besides, one of the real issues is the use, in most of the studies reported, of FBS for the expansion of ASCs. Indeed, it is well known that this approach is not suitable for clinical use of ASCs or their secretome, due to the risk of transmission of disease as well as xenogeneic immune reactions in the transplanted host [33,86]. In this regard, recently, human serum albumin (HSA), human serum (HS), or knockout serum replacement (KSR) have been suggested as useful replacements of FBS for ASC cryopreservation and clinical banking [87]. It is therefore essential to carry out studies using a human serum or synthetic supplementation, which could also be certified for good manufacturing practice (GMP) use of ASCs or their secretome for wound healing. Moreover, taking into account the minimal information for studies of extracellular vesicles (MISEV) 2018 guidelines and a recent review on this topic [72,88], it is also necessary to carry out studies aimed at comparing the two main techniques of preparation of EVs: Ultracentrifugation and precipitation. At present, based on the available literature data, considering the multiple variables in the studies conducted, it is not possible to notice any differences between the two EV preparation procedures regarding the quality and/or efficacy in wound healing models. However, while a comparative analysis of the results obtained by the different groups is difficult due to the vast diversity of experimental conditions, the available findings are undoubtedly encouraging and actively support the use of cell-free therapies for the treatment of chronic non-healing wounds. Taken together, the results of in vitro studies could contribute to a better understanding of the biomolecular mechanisms underlying the influence of ASC-secretome on cell types associated with the wound healing process and provide a basis for further and more targeted investigations that are useful to addressing the ways of accelerating chronic non-healing wound closure. Similarly, the described experimental in vivo studies, with the objective of showing the pro-wound healing potential of ASC-CM or ASC-EVs, were conducted on some animal models and with different techniques to create the skin lesions. Thus, as for in vitro studies, the framework of in vivo experimental studies also shows some variability at all levels of experimental design, methodologies, and applied protocols (e.g., animal models of wound healing, methods of preparation of ASC-CM or ASC-EVs, concentrations of CM or EVs used in vivo, delivery routes). So, to further demonstrate the clinical relevance of cell-free therapies, more homogeneous in vivo studies in animal models are required to better define the effects of ASC-secretome on different phases of cutaneous wound repair. In this regard, the recent article of Grada et al. [89] appears particularly useful because it exhaustively describes the advantages and disadvantages of animal models of acute healing or impaired healing developed to study the intricated cellular and biochemical processes of wound repair, and to assess the efficacy and safety of potential innovative therapies. The authors, while aware of the importance of animal models and the valuable information they can provide us, rightly emphasize the importance of knowing the peculiarities of each animal model to assess its merits and limitations based on the experimental objectives. Given the complexity, variety, and multifactorial framework of chronic wounds in humans, identifying an ideal animal model for the study of non-healing chronic wounds is undoubtedly an ambitious goal to achieve. This is also because of the multiple intrinsic and extrinsic factors that can strongly affect wound repair, including, among others, impaired circulation, chronic inflammation, nutrition, aging, limited physical activity, and chronic diseases. The hope is that, given the incredible progress in manipulating mice and other animals, new models of chronic non-healing wounds will be defined to better assess the benefits of new promising therapies, including cell-free ASC-CM or ASC-EV therapy (Figure 2).

## Figures and Tables

**Table 1:** Effects of ASC-Conditioned Medium/Secretome on Wound Healing: In vitro studies.

ASC Source	Model	Effect	Reference
Human lipoaspirates from healthy females.	HDF	Increased cell proliferation and migration. Higher type I collagen secretion.	[56]
Human lipoaspirates from female patients (18–35 years old).	HDF	Increased cell proliferation and migration.	[57]
Human adipose tissue from donors undergoing abdominoplasty.	HDF	No effect.	[58]
	HUVEC	No effect.	
	Human keratinocytes	Reduction of keratinocyte proliferation.	
Human abdominal adipose tissue and lipoaspirates from patients undergoing panniculectomy or abdominoplasty.	3-D skin cultures of primary human keratinocytes	Acceleration of wound closure.	[59]
Commercial human ASCs.	HUVEC	Increased cell proliferation, migration, and invasion ability.	[60]
	HaCaT	Increased cell proliferation, migration, and VEGF secretion.	
Commercial human ASCs of normal donors (25–45 years old) undergoing elective surgery.	HDF	Increased cell migration.	[61]
Adipose tissue of breast cancer patients.	HDF	Increased cell proliferation, migration, and invasion ability.	[46]
	HEKa		
	HUVEC		
Human sub-cutaneous adipose tissue from male patient (51-year-old) undergoing skin-graft surgery.	HDF	Increased cell proliferation and migration.	[62]
	HaCaT		
Sub-cutaneous adipose tissue (average age of patients 47.2 ± 10.8) or sub-cutaneous lipoma (average age of patients 41.8 ± 7.1).	RAW 264.7	Functional cell activation and M2 phenotype polarization.	[64]
	L929 Fibroblasts	Acceleration of scratched monostrate closure, increased cell migration.	

HDF: human dermal fibroblast; HUVEC: human umbilical vein endothelia cell; ASCs: adipose tissue-derived stem cells; HaCaT: spontaneously immortalized human keratinocyte cell line; VEGF: vascular endothelial growth factor; HEKa: human epidermal keratinocyte, adult.

**Table 2:** Effects of ASC-conditioned medium/secretome on wound healing: In vivo studies.

ASC Source	Model	Effect	Reference
Human sub-cutaneous adipose tissue.	Rat skin excisional wound model	Stimulation of wound closure, angiogenesis, proliferation, and macrophage infiltration.	[65]
Commercial rat ASC.	Rat skin excisional wound model	Accelerated wound closure, increased macrophage recruitment and M2 phenotype polarization.	[67]
Adipose tissue of breast cancer patients.	Nude mice full-thickness excisional skin wound model	Stimulation of wound closure, dermal thickening, angiogenesis, and immune cell recruitment.	[46]
Commercial human ASCs of normal donors (25–45 years old) undergoing elective surgery.	Rat ischemic skin wound model	Acceleration of wound closure.	[61]
Gluteal region of Yorkshire pigs.	Yorkshire pig diabetic skin wound model	Acceleration of wound closure, increased angiogenesis, reduced inflammatory profile.	[68]

ASCs: adipose tissue-derived stem cells.

**Table 3:** Effects of ASC-secreted extracellular vesicles on wound healing: In vitro studies.

ASC Source	Model	Effect	Reference
Commercial human ASC	HMEC	Stimulation of vessel-like structure formation	[73]
Human sub-cutaneous adipose tissue obtained from healthy females (18–35 years old).	HDF	Stimulation of cell migration and proliferation. Higher collagen synthesis.	[74]
Human sub-cutaneous adipose tissue of normal donors (25–45 years old) undergoing elective surgery.	HDF	Increase of cell migration rate.	[61]
Adult fat samples.	HUVEC	Stimulation of angiogenesis, upregulation and downregulation of angiogenesis-stimulating or -inhibitory genes, respectively.	[75]
Human lipoaspirates.	Primary human keratinocytes HDF	Increased cell proliferation and migration rate. Upregulated Akt pathway	[76]
Human Lipoaspirates (mean age of the patients: 20 ± 1.5 years).	HDF	Stimulation of cell proliferation and migration. Increased type-1 and type-3 collagen levels. Upregulated PI3K/Akt signaling pathway.	[78]
Human subcutaneous adipose tissue.	HUVEC	Increase of cell proliferation, migration and angiogenesis. Upregulation of proliferative markers and growth factors. Upregulation of Akt and ERK signaling pathways.	[79]
	HaCaT	Increase of cells migration and proliferation. Upregulation of proliferative markers and growth factors. Upregulation of Akt and ERK signaling pathways.	
	Primary human foreskin fibroblasts	Increase of cells migration and proliferation	
		Upregulation of proliferative markers and growth factors.	
		Upregulation of Akt and ERK signaling pathways.	
Human lipoaspirates.	HDF	Stimulated cell proliferation and migration. Increased expression of genes related with skin regeneration.	[80]
Adipose tissue harvested from healthy people or normal rats.	Endothelial progenitor cells isolated from peripheral blood of healthy subjects or patients with diabetes mellitus.	Increased cell viability and tube formation ability.	[81]
Human facial adipose tissue.	HaCaT	Increased cell proliferation and migration. Reduced H_2_O_2_-induced apoptosis levels.	[83]

ASC: adipose tissue-derived stem cell; HMEC: human mammary epithelial cell; HDF: human dermal fibroblast; HUVEC: human umbilical vein endothelial cell; Akt: protein kinase B; PI3K: phosphoinositide 3-kinase; ERK: extracellular signal-regulated kinase; HaCaT: spontaneously immortalized human keratinocyte cell line.

**Table 4:** Effects of ASC-secreted Extracellular Vesicles on Wound Healing: In vivo studies.

ASC Source	Model	Effect	Reference
Human subcutaneous adipose tissue obtained from healthy females (18–35 years old).	Mouse full-thickness skin wound model	Acceleration of wound repair.	[74]
Human subcutaneous adipose tissue obtained after liposuction surgery (mean age of the patients: 20 ± 1.5 years).	Mouse full-thickness skin wound model	Increased number of blood vessels. Acceleration of wound repair.	[78]
Human subcutaneous adipose tissue obtained by liposuction.	Rat excisional wound-splinting model.	Acceleration of wound repair.	[76]
Human subcutaneous adipose tissue.	Mouse full-thickness skin wound model	Acceleration of wound repair. Increased collagen deposition. Increased neovascularization and cell proliferation.	[79]

## Abbreviations

MMP	Metalloproteinase
ECM	Extracellular matrix
MSCs	Mesenchymal stem cells
ASCs	Adipose tissue-derived stem cells
CM	Conditioned medium
EVs	Extracellular vesicles
HDFs	Human dermal fibroblasts
HUVECs	Umbilical vein endothelial cells
MVs	Microvesicles
